# Metabolomics reveals perturbations in endometrium and serum of minimal and mild endometriosis

**DOI:** 10.1038/s41598-018-23954-7

**Published:** 2018-04-24

**Authors:** Mainak Dutta, Brajesh Singh, Mamata Joshi, Debanjan Das, Elavarasan Subramani, Meenu Maan, Saikat Kumar Jana, Uma Sharma, Soumen Das, Swagata Dasgupta, Chaitali Datta Ray, Baidyanath Chakravarty, Koel Chaudhury

**Affiliations:** 1 School of Medical Science and Technology, Indian Institute of Technology, Kharagpur, West Bengal India; 2 Department of Biotechnology, Birla Institute of Technology and Science, Pilani (Dubai Campus), Dubai, United Arab Emirates; 3 National Facility for High-field NMR, Tata Institute of Fundamental Research, Mumbai, Maharashtra India; 4 School of Biotechnology, Jawaharlal Nehru University, New Delhi, Delhi, India; 5 Department of Chemical and Bio-Technology, National Institute of Technology, Arunachal Pradesh, India; 6 Department of N.M.R., All India Institute of Medical Sciences, New Delhi, Delhi India; 7 Department of Chemistry, Indian Institute of Technology, Kharagpur, West Bengal India; 8 Institute of Post Graduate Medical Education & Research, Obstetrics & Gynecology, Kolkata, West Bengal India; 9 Institute of Reproductive Medicine, Sector-III, Kolkata, West Bengal India; 10 Department of Electronics & Communication Engineering, DSPM-IIIT, Naya Raipur, CG India

**Keywords:** Diagnostic markers, Diagnostic markers

## Abstract

Endometriosis is a common benign gynecological disease, characterized by growth and proliferation of endometrial glands and stroma outside the uterus. With studies showing metabolic changes in various biofluids of endometriosis women, we have set upon to investigate whether endometrial tissue show differences in their metabolic profiles. ^1^H NMR analysis was performed on eutopic endometrial tissue of women with endometriosis and controls. Analysis was performed on spectral data and on relative concentrations of metabolites obtained from spectra using multivariate and univariate data analysis. Analysis shows that various energy, ketogenic and glucogenic metabolites have significant altered concentrations in various stages of endometriosis. In addition, altered tissue metabolites in minimal and mild stages of endometriosis were explored in serum of these patients to assess their role in disease diagnosis. For Stage I diagnosis alanine was found to have 90% sensitivity (true positives) and 58% specificity (true negatives). For Stage II diagnosis alanine, leucine, lysine, proline and phenylalanine showed significant altered levels in serum. While sensitivity of these serum metabolites varied between 69.2–100% the specificity values ranged between 58.3–91.7%. Further, a regression model generated with this panel of serum markers showed an improved sensitivity and specificity of 100% and 83%, respectively for Stage II diagnosis.

## Introduction

Endometriosis is an estrogen dependent benign gynecological disorder and is defined by the presence of endometrial tissue consisting of glands and stroma outside the uterine cavity; commonly on the pelvic peritoneum, ovaries, and rectovaginal septum. Rarely, extra pelvic sites such as thorax^1^, cutaneous tissues^2^, bone^3^, and even the brain^4^ are also found to have infiltrating endometrial tissue growth. It is one of the most common causes of female infertility of unknown etiology which affects menstruating women in their reproductive age and may result from imbalance of the cellular equilibrium^5^. It affects 6–10% of women in their reproductive age, 50–60% adult and teenage women with complaints of pelvic pain and upto 50% of women reporting for infertility^6^. The symptoms of the disease include dysmenorrhoea, dyspareunia, dysuria and chronic abdominal pain which often appear late and overlap with other gynecological conditions such as pelvic adhesions, pelvic inflammatory disease, adenomyosis, ovarian cysts, and non-gynecological conditions such as inflammatory bowel syndrome, intestinal cystitis, irritable bowel syndrome etc^6^; often making diagnosis challenging. Currently, several diagnostic methods are available including ultrasonography and magnetic resonance imaging, nevertheless the gold standard for conclusive diagnosis remains surgical assessment of the pelvis by laparoscopy followed by biopsy^7^. Though CA-125 is the most popular biomarker for non-invasive diagnosis of endometriosis, several repeated studies have shown that CA-125 may be of more benefit in diagnosing Stages III–IV (advanced), than Stages I–II (mild)^8^.

Metabolomics is now increasingly being recognized as a powerful tool for elucidating pathogenesis and disease biomarker identification. This new ‘omics’ approach is used to perform global analysis of low molecular weight metabolites in various biological samples. An untargeted metabolomics approach provides an unbiased view of global metabolite changes in biological samples. The approach is hypotheses generating and exploratory in nature thereby helping in generating novel hypothesis or support older findings. Mass spectrometry (MS) and nuclear magnetic resonance (NMR) based platforms are the two most widely used analytical tools used for metabolomics studies. Unlike NMR, MS-based metabolomics provides better sensitivity for metabolomics research. With a combination of different ionization techniques and mass analyzers MS provides wide detection coverage of metabolites. NMR spectroscopy, on the other hand, is reproducible, quantitative and does not require extra steps for sample preparation, such as separation or derivatization^9^.

Despite several studies on metabolomics in various diseases, use of this approach is still sparse in endometriosis^10–12^. NMR and MS based metabolomics studies on endometrial fluid^13^, follicular fluid^14^, urine^10^, serum^15^ and plasma^16^ of endometriosis women have shown remarkable changes in their metabolite profile. Since endometrial tissue communicates with the blood directly or through extracellular fluids, several metabolites may be secreted into the blood stream. A circulatory diagnostic marker originating from the affected tissue is expected to be more reliable. The present study investigates ^1^H NMR based metabolic profiling of endometrial tissue to obtain a comprehensive idea of the metabolic perturbations associated with disease development and progression. The levels of altered tissue metabolites were further investigated in serum of paired endometriosis patients for their role in mild stage of the disease diagnosis.

## Material and Methods

### Subject selection

Necessary approvals were obtained from the Institutional Research Ethics Board of the Institute of Reproductive Medicine, Kolkata (IRM/IEC/BNC-IHP dated 30.11.2013); Institute of Post Graduate Medical Education & Research, Kolkata (Inst/IEC/551 dated 15.01.2014) and Indian Institute of Technology, Kharagpur, India (IIT/SRIC/AR/2013 dated 20.02.2013) prior to commencement of the study. All methods and experiments were performed in compliance and accordance to relevant guidelines and regulations. Endometriosis subjects were recruited from women presenting with endometriosis-associated symptoms and subsequently confirmed with laparoscopy followed by biopsy. Patients with age ≥40 years, ovarian tumors, polycystic ovarian syndrome, tubal block, anatomical abnormalities of the uterus, hydrosalphinx, pelvic inflammatory disease, other pelvic pathological conditions, leiomyoma, etc., were excluded from the study. Also, women who had received medical/hormonal supplementation during the last 3 months were excluded. 95 patients met the inclusion and exclusion criteria and were confirmed to have endometriosis. Staging was performed according to the revised American Society for Reproductive Medicine (initially named as AFS)^17^ criteria with Stage I classified as minimal (n = 20), Stage II as mild (n = 13), Stage III as moderate (n = 17) and Stage IV as severe endometriosis (n = 45).

Twenty four healthy fertile subjects were recruited from patients undergoing interval tubectomy (sterilization) and classified as controls. Women who had their last delivery during the 2–3 preceding years and having normal tubal pathology were included in the study. Exclusion criteria such as women ≥40 years of age and women receiving medical/hormonal supplementation during the last 3 months were considered while selecting control subjects. Blood and tissue samples were collected from both the study and control groups during mid secretory phase. Blood was drawn before anesthesia. Clinical information associated with each sample group is summarized in Table 1. Prior written informed consent was taken from all participants.

**Table 1:** Clinical characteristics of samples included in the study.

Characteristic (Tissue)	Healthy women (n = 24)	Endometriosis patients (n = 95)
Origin	Eastern region of India and Bangladesh	Eastern region of India and Bangladesh
Age (y), mean ± SD	28.42 ± 3.2	29.37 ± 5.8
BMI (kg/m^**2**^), mean ± SD	26.17 ± 1.9	25.99 ± 1.5
***Stage of the disease***		
Stage I (Minimal)	—	20
(Stage II) (Mild)	—	13
(Stage II)I (Moderate)	—	17
Stage IV (Severe)	—	45

### Sample collection

Endometrial tissue was snap frozen and stored at −80 °C till further use. Blood samples were collected from women with endometriosis and controls, as mentioned previously^15^.

### Sample preparation and metabolite extraction

#### Tissue

Metabolite extraction from tissue was performed according to Beckonert and coworkers^18^. Briefly, 100 mg of frozen tissue was weighed and grinded in a liquid nitrogen cooled mortar with 6% ice-cold perchloric acid. The sample was then vortexed and placed on ice for 10 min followed by centrifugation for 10 min at 12,000 g at 4 °C. The supernatant was then neutralized to pH 7.4 with 2 M potassium carbonate and left on ice for 30 min to precipitate the potassium perchlorate salts. The sample was then finally centrifuged, supernatant freeze–dried and stored at −80 °C. Before NMR acquisition, the tissue extracts were resuspended in 650 μl NMR buffer (100 mM sodium phosphate buffer, pH 7.4, in deuterated water (D_2_O), containing 1 mM sodium salt of 3-(trimethylsilyl) propionic-2,2,3,3,d4 acid (TSP). The samples were vortexed, centrifuged at 12,000 g for 5 min and finally 600 μl of the supernatant was transferred into an NMR tube.

#### Serum

Sample preparation for NMR analysis was performed as mentioned in our earlier study^15^. Briefly, serum samples were thawed and homogenized using a vortex mixer prior to NMR analysis. 200 µl of serum was mixed with 400 μl D_2_O containing 1 mM TSP. Following centrifugation (8000 rpm, 5 min), 600 µl of each sample was transferred into NMR tubes

### ^1^NMR data acquisition

NMR experiments were performed as explained in our earlier study^15^. Briefly, proton NMR spectra were recorded at 300 K using a 700 MHz Bruker Avance AV III spectrometer. CPMG spin-echo spectra were recorded with spectral width of 14 000 Hz and an acquisition time of 0.58 s. The pulse sequence used included a solvent pre-saturation routine for the suppression of the water signal. The resulting spectra were phased and baseline corrected, and chemical shifts referenced to TSP (δ = 0) by Bruker TOPSPIN 2.1 software. Normalization of spectrum to its total area was performed using MestReNova version 7.1.0 to limit the effect of concentration differences between samples. Minor chemical shifts due to pH alterations in tissue extracts and serum were adjusted and aligned using *i*coshift algorithm tool for MATLAB^19,20^. Individual metabolites were manually identified based on expected chemical-shift and coupling-constant values obtained from various sources, including earlier published articles, literatures and cross checked from the Human Metabolome Database (HMDB). Further, peak assignment was validated with COSY (Correlation spectroscopy) and TOCSY (Total correlation spectroscopy) spectra.

### Multivariate analysis

Multivariate data analysis was performed on the full tissue spectra from 0.5–10 ppm in SIMCA 13.0.2 (Umetrics, Sweden). Water region from 4.5–5.15 ppm was excluded from the analysis. Unit variance scaling was applied to the data matrix to give equal weightage to all the variables. Following normalization and scaling, both the tissue and serum dataset was subjected to unsupervised and supervised multivariate analysis separately. Principal component analysis (PCA) was performed to detect inherent clustering and outliers in an unbiased manner. Partial least square discriminate analysis (PLS-DA) and a better classification model, orthogonal PLS-DA (OPLS-DA) was used to maximize class separation. Variables not relevant to class separation get removed in the OPLS-DA model. Therefore, only one predictive component is used for the discrimination between two classes^21,22^. To avoid over-fitting of the supervised models, the default 7-round cross-validation procedure in SIMCA software package was used. The reliability of the models was evaluated by the parameters R2 and Q2. R2 represents goodness of fit indicating explained variation in the dataset, whereas the cross validation parameters Q2 represent predictability of the model. Further, CV-ANOVA (analysis of variance testing of cross-validated predictive residuals) was carried out to ascertain the statistical significance of the OPLS-DA models. Significant variables were identified using S-line correlation plot.

### Univariate analysis

Univariate analysis was performed both for tissue and serum spectra. The statistically significant variables extracted were subjected to spectral integration using MestReNova version 7.1.0 s. Baseline corrections were carried out wherever necessary. Non-parametric Kruskal–Wallis test was used with the Dunn multiple comparisons test to identify the statistically significant metabolites. Spearman rank correlation analysis was performed between tissue metabolite levels and rAFS score of women with endometriosis to ascertain the role of statistically significant metabolites in disease progression. Spearman rank correlation analysis between serum and tissue was performed for the statistically significant serum metabolites in minimal/mild endometriosis cases. GraphPad Prism version 5.0 (GraphPad Software, San Diego California, USA) was used for these univariate analyses. Receiving operating characteristic (ROC) curve analysis for diagnostic potential study was performed using MedCalc software (version 12.2.1.0).

## Results

Several metabolites from diverse chemical classes could be identified from the ^1^H NMR spectra of endometrial tissue samples. These metabolites included amino acids (valine, glycine, alanine, leucine, proline, isoleucine, glutamate, glutamine, aspartate, asparagine, threonine, tyrosine, phenylalanine, ornithine, creatine), organic acids (citrate, 3-hydroxybutyric acid, lactate, acetate, succinate, formate, taurine, nicotinurate), nucleosides/nucleotides (inosine, adenine, uracil, uridine) and sugars (glucose, *myo*-inositol, glycogen). The list of metabolites along with their chemical moieties and corresponding chemical shifts are provided in Supplementary Table 1. A representative ^1^H-NMR spectrum of endometrial tissue from an endometriosis patient and the assignment of the representative metabolites is provided in Supplementary Figure 1.

### Multivariate analysis

**Figure 1:**
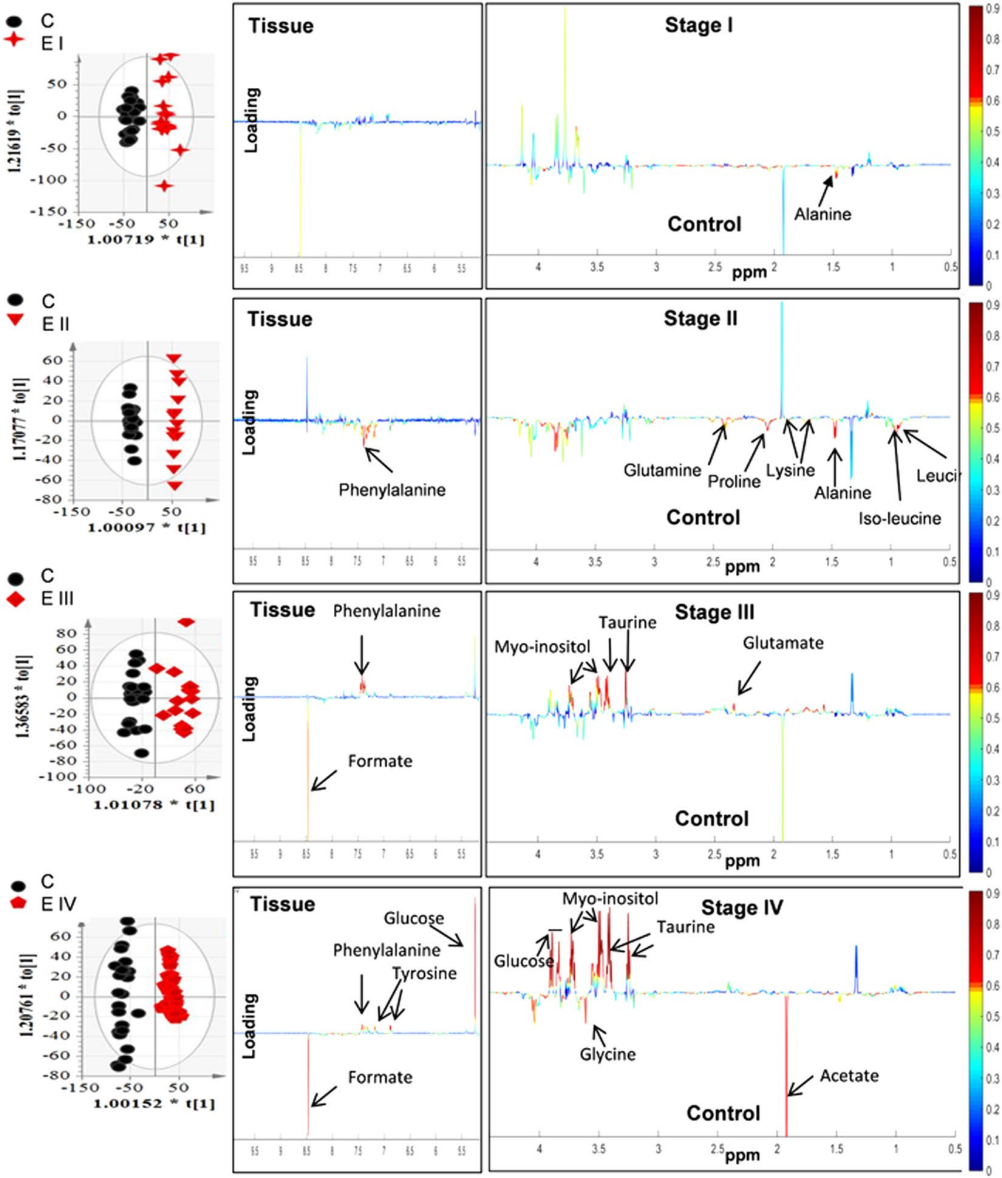
OPLS-DA scores (left) and loadings plots for multivariate analysis with tissue samples collected from controls (n = 24) and stage I (n = 20), II (n = 13), III (n = 17) and IV (n = 45) of endometriosis patients. Results were from the 7-fold cross-validated models and colored scales were for the correlation coefficients (|r|) of variables.

PCA analysis of tissue NMR spectra revealed clustering among the various stages of endometriosis and controls (Supplementary Figure 2). Supervised models such as PLS-DA (Supplementary Figure 2) and OPLS-DA (Fig. 1) showed improved discrimination with good model prediction parameters (Supplementary Table 2).

### Identification of statistically significant metabolites

**Figure 2:**
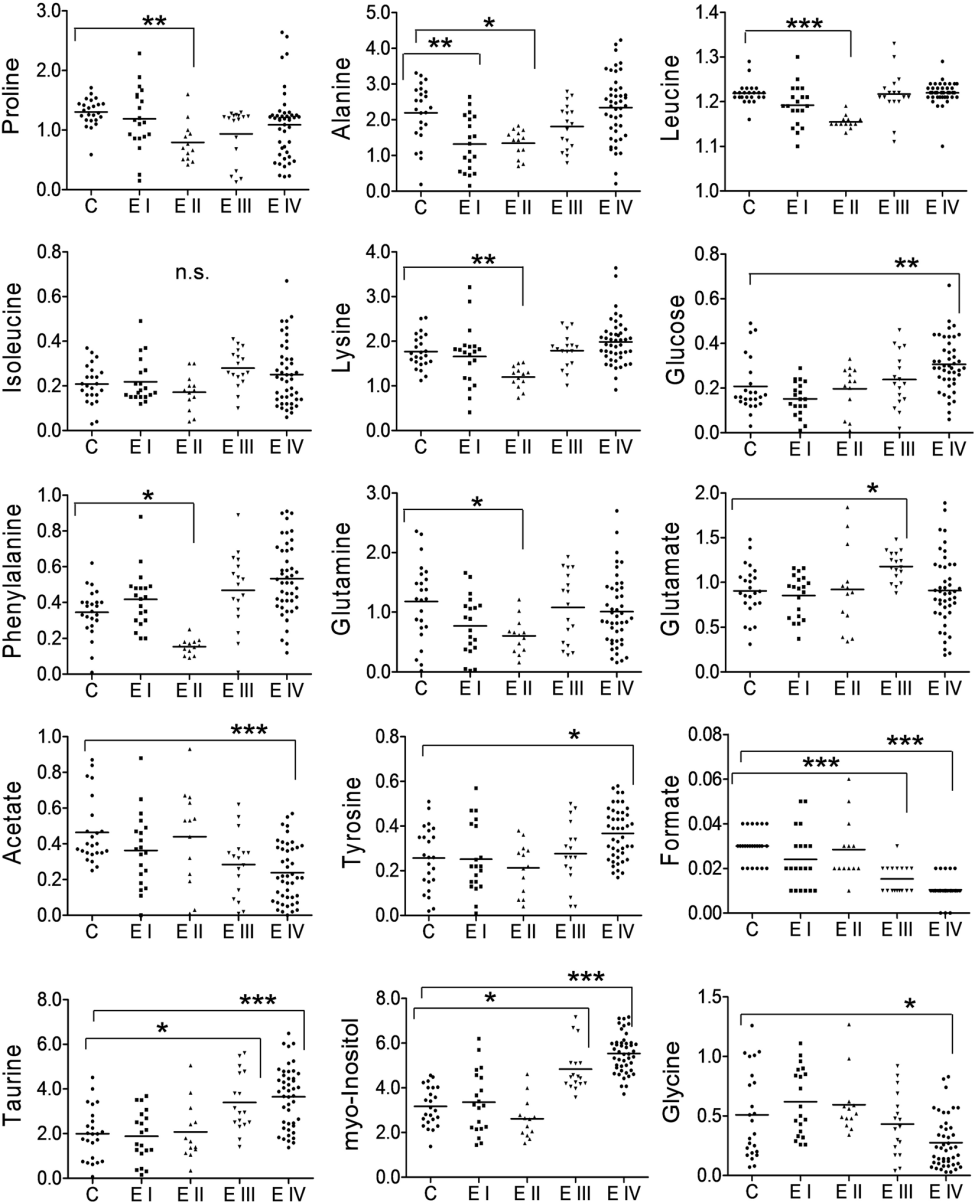
Results of metabolite integral values for selected tissue metabolites. Horizontal lines represent group means. Kruskal-Wallis test was used with the Dunn multiple comparisons test. Levels of statistical significance are as follows: *p < 0.05; **p < 0.01; ***p < 0.001.

**Figure 3:**
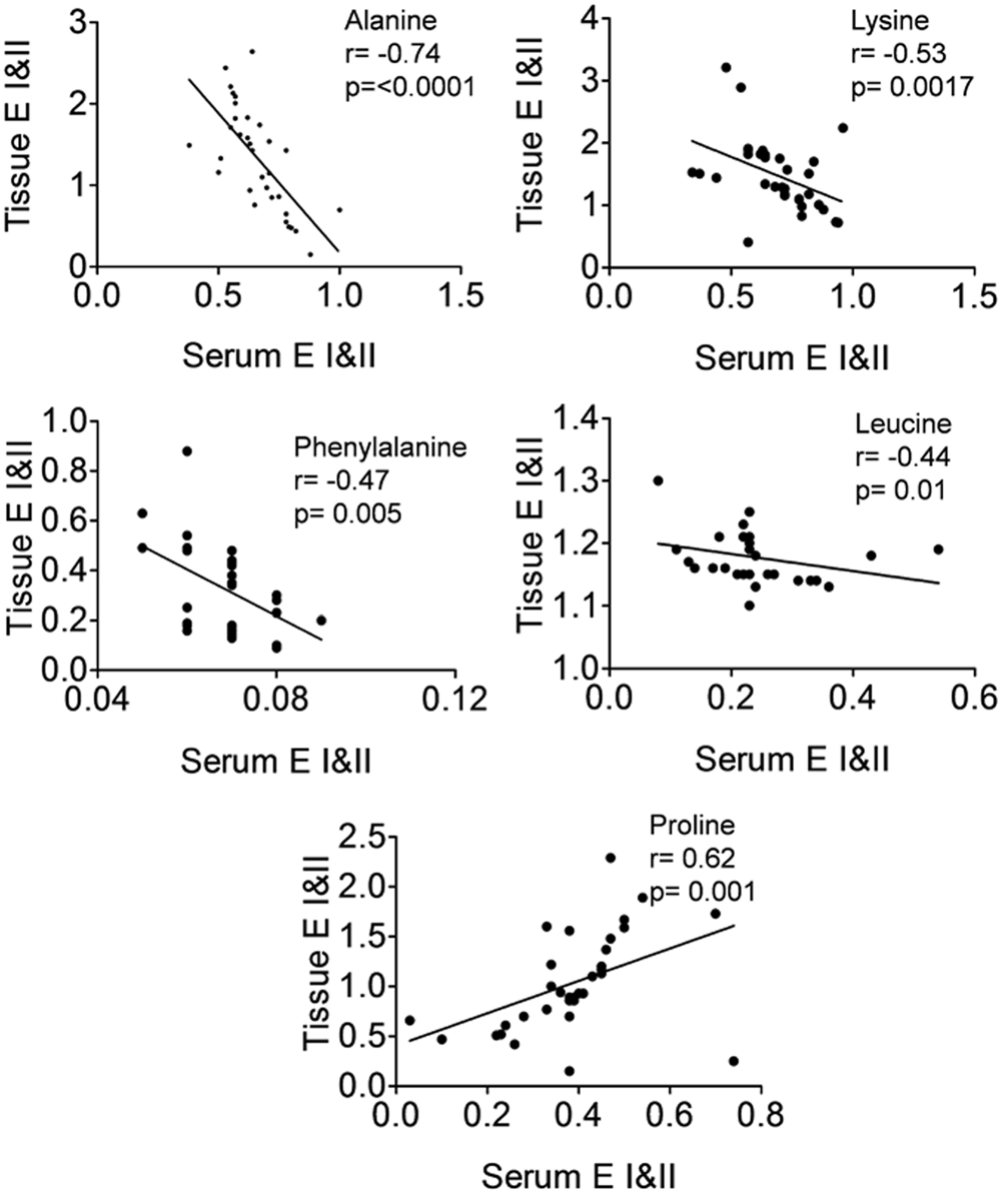
Results of correlation analysis between tissue and serum metabolite expression levels. Non-parametric Spearman correlation test was used.

**Figure 4:**
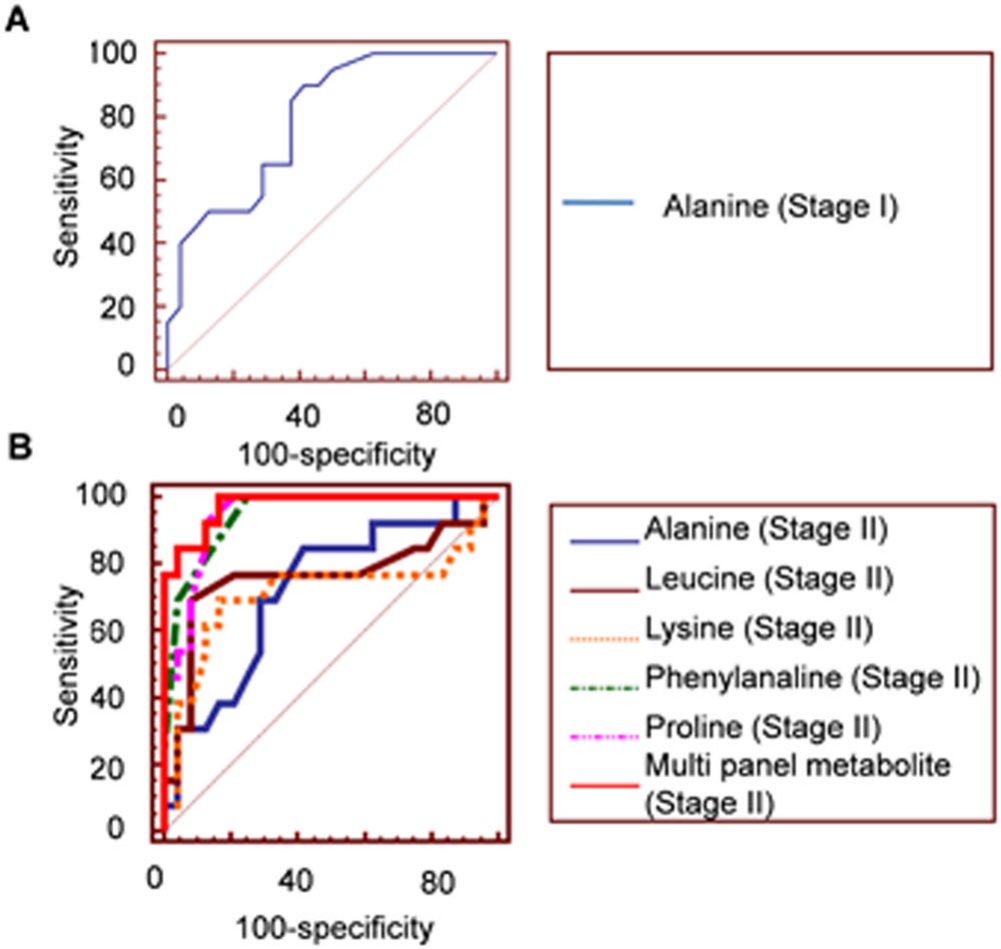
ROC curve analysis of the identified serum metabolite markers for (**A**) Stage I and (**B**) Stage II diagnosis.

A two variable extraction procedure was used to extract the most relevant tissue metabolites responsible for disease classification. First, S-line correlation plot was used to identify the most significant variables responsible for class separation in tissue (Fig. 1). The variables with higher values of correlation coefficient (denoted by red color) were the major metabolites contributing maximum towards class separation. These metabolites were then subjected to relative quantitative analysis using their individual integral values. Metabolite such as isoleucine which was found to be significant in the first step is now ruled out from the list of relevant metabolites, owing to their insignificant p value during univariate analysis (Fig. 2). Further, the statistically relevant tissue metabolites in early (minimal/mild) endometriosis were identified in serum NMR spectra and subjected to relative quantitative analysis using their individual integral values. The relevant tissue and serum metabolites were subjected to correlation analysis. While alanine, lysine, phenylalanine and leucine showed negative correlation, proline showed positive correlation (Fig. 3). The diagnostic potential of these markers were assessed in serum of minimal and mild endometriosis patients (Table 2 and Fig. 4). A partial least squares (PLS) regression model with a combination of the panel of serum markers followed by ROC analysis showed improved sensitivity and specificity of 100% and 83% for Stage II diagnosis.

**Table 2:** Diagnostic potential of the identified serum markers.

Metabolite	Sensitivity (%)	Specificity (%)
Alanine (Stage I)	90	58.3
Alanine (Stage II)	84.6	58.3
Leucine (Stage II)	69.2	91.7
Lysine (Stage II)	69.2	83.3
Proline (Stage II)	92.3	87.5
Phenylalanine (Stage II)	100	75
Multi panel metabolite (Stage II)	100	83

## Discussion

Though it is now widely accepted that metabolomics promises immense potential understanding pathogenesis, diagnosis and therapy monitoring of many diseases; use of this approach in endometriosis is still at its nascent stage. This approach, nevertheless, has gained momentum, as evident by three studies investigating endometriosis-associated metabolic changes in serum^15,16^ and urine samples^10^. We here study the metabolite changes in endometrial tissue of women with endometriosis (Stage I–IV) using ^1^H NMR based metabolomics. Since CA-125 is routinely used and is quite efficient in diagnosis of stage III & IV of endometriosis, our subsequent objective was to identify a panel of metabolites which can be used for non-invasive diagnosis of Stage I & II of the disease. We, therefore, investigated the levels of tissue metabolites in serum which were found to be relevant to Stage I & II disease pathology. To the best of our knowledge this is the first report on endometrial tissue metabolite profile of women with endometriosis.

Although the precise mechanism of disease development remains unknown, according to Sampson’s hypothesis, endometriosis is established when retrograde endometrial cells implant within the pelvic cavity. It is now widely accepted that there are fundamental abnormal changes within the eutopic endometrium of women with endometriosis compared to normal endometrium of healthy women. Several studies have shown that eutopic endometrium has enhanced ability of proliferation, angiogenesis, and implantation and, thus, greater probability of escaping the unfavorable conditions of the ectopic environment. Therefore, the character of eutopic endometrium determines the fate of the backward-flowing endometrial tissue in the pelvic cavity^23^. The present study, therefore, attempts to study the metabolic changes in eutopic endometrium of women with endometriosis. It is well accepted that endometriosis exhibits features similar to that of malignancy^24,25^. Though a benign condition, endometriosis appears to act like cancer characterized by cell invasion, uncontrolled growth, neo-vascularization and a decrease in the number of cells due to apoptosis^26^. Increasing evidences suggest that markers for these tumorigenic properties are also expressed in eutopic tissue^27–30^ indicating that endometrium in these patients may already have been affected before they migrate to the peritoneum resulting in endometriosis^31^; thus supporting the retrograde menstruation hypothesis. Taurine is a β-amino acid which is found abundantly in various mammalian tissues, and its concentration has been found to be increased in several tumors^32–34^. Elevated taurine levels in tumors are associated with increased proliferative activity and cell density^35^. Another metabolite, *myo*-Inositol, is correlated with tumor cell density^36^. While its level is reported to be downregulated in cancer with a negative correlation with taurine^37,38^, several other studies suggest increased levels of both *myo*-inositol and taurine in tumors of the prostate and squamous cell carcinoma^39,40^. Following the analogy of endometriosis with the neoplastic process, increased levels of these two metabolites in the tissue of advanced stages of endometriosis (Figs 1 and 2) is associated with the higher proliferative activity seen in endometriotic lesions. Further, both taurine and *myo*-inositol showed strong positive correlation with rAFS scores (Supplementary figure 3) suggesting their role in disease progression. Also, our observation of higher taurine levels in endometriosis women is in agreement with an earlier metabolomics study performed on urine samples of endometriosis patients^10^. Endometriosis is associated with angiogenesis^41^ which is further associated with elevated de novo nucleotide synthesis^42^. Formate is an integral metabolite of purine biosynthesis. Owing to increased angiogenesis and tissue growth in women with advanced stages of endometriosis, it is likely that the formate molecules are incorporated into the purine nucleotide synthesis leading to its depletion. This may be the possible explanation for decreased formate levels observed in endometrial tissue of women with severe endometriosis.

Several amino acids were found to be dysregulated in tissue of various stages of endometriosis women compared to healthy controls. Alteration in glutamine and glutamate level was observed in Stage II and Stage III of endometriosis patients. Both glutamine and glutamate are glucogenic amino acids and are considered to be vital for maintenance of cell function^43^. They can be used for a diverse array of processes^44^ according to the demands of the body during severe illness. A recent study has infact associated altered glutamine and glutamate levels to endometriosis-associated pelvic pain^45^. A number of amino acids including alanine, lysine, phenylalanine and leucine showed significantly lower levels in endometrial tissue of women with endometriosis relative to healthy controls. This may be due to the fact that endometriosis results from physiological mechanism of tissue injury and repair^46^. The catabolic state induced in response to injury in endometriosis leads to increased breakdown of endogenous protein and release of free amino acids in circulation. This is further supported by our results where we observed an inverse relationship between tissue and serum levels for alanine, lysine, phenylalanine and leucine. Another amino acid, proline was also found to have significant lower levels in tissue, but showed positive correlation with serum level of Stage II endometriosis. In advanced stages of the disease, though not statistically significant, a general downward trend was observed in tissue samples (Fig. 1). A recent report proposes that the degradation of proline occurs only through proline dehydrogenase and is associated with reactive oxygen species (ROS) generation^47^. With increasing evidence suggesting that ROS may be involved in endometriosis progression^48^, it may be worthwhile to further explore the role of proline in the pathogenesis of the disease.

Tissue metabolite levels may or may not be reflected in circulation. For study of diagnostic potential of the early stage (Stage I/II) dysregulated tissue metabolites, their levels were studied in serum of paired samples. It was encouraging to find that five of the dysregulated tissue amino acids were also found to have altered levels in serum of these patients. Interestingly, while tissue showed lower levels for alanine, lysine, phenylalanine and leucine, serum samples showed an inverse relationship for these metabolites. It was, therefore, not surprising to find a significant negative correlation between tissue and serum levels of alanine, lysine, phenylalanine and leucine (Fig. 3). A positive correlation was observed for proline. Further, for minimally invasive diagnosis, the diagnostic potential of these five metabolites was assessed in serum. For Stage I diagnosis alanine was found to have 90% sensitivity (true positives) and 58% specificity (true negatives). For Stage II diagnosis maximum sensitivity was observed for phenylalanine, however its specificity was found to be poor. Leucine showed maximum specificity of 91.7%, however its sensitivity was again found to be poor (69.2%). It is now being widely recognized that a panels of markers would have better diagnostic potential compared with individual markers^49^. This is in accordance with our findings for Stage II diagnosis where we find sensitivity and specificity of 100% and 83%, respectively for the PLS model developed using the combined panel of markers.

## Conclusion

In summary, we have for the first time identified characteristic metabolites in eutopic endometrial tissue of various stages of endometriosis. The metabolic perturbations mediated by the metabolites which can significantly discriminate between endometriosis and controls are discussed. Further, the relevant tissue metabolites are correlated with the corresponding serum metabolites in early stage of the disease for investigating their role in early diagnosis. Five serum amino acids, proline, alanine, lysine, phenylalanine and leucine were found to be suitable for endometriosis diagnosis. Future studies will focus on the validation of these results in larger cohorts of patients. Also, a follow-up study to track the levels of the metabolites from the onset of endometriosis could help improve its clinical management. In addition, inclusion of additional patient groups with other conditions associated with pelvic inflammation will help in deciphering the cross-talk between endometriosis and associated pelvic inflammation. It is also important to mention that for robust assessment of these markers, their expression levels in endometriosis patients receiving hormone medications should also be investigated. Furthermore, there is a need to compare the present findings, which are restricted to the secretory phase, to the candidate metabolites’ expression in the proliferative phase for developing a comprehensive diagnostic test for endometriosis.

## Electronic supplementary material

Supplementary Information
