# Author Correction: Metabolomics reveals perturbations in endometrium and serum of minimal and mild endometriosis

**DOI:** 10.1038/s41598-020-59776-9

**Published:** 2020-02-12

**Authors:** Mainak Dutta, Brajesh Singh, Mamata Joshi, Debanjan Das, Elavarasan Subramani, Meenu Maan, Saikat Kumar Jana, Uma Sharma, Soumen Das, Swagata Dasgupta, Chaitali Datta Ray, Baidyanath Chakravarty, Koel Chaudhury

**Affiliations:** 1 School of Medical Science and Technology, Indian Institute of Technology, Kharagpur, West Bengal India; 2 Department of Biotechnology, Birla Institute of Technology and Science, Pilani (Dubai Campus), Dubai, United Arab Emirates; 3 National Facility for High-field NMR, Tata Institute of Fundamental Research, Mumbai, Maharashtra India; 4 School of Biotechnology, Jawaharlal Nehru University, New Delhi, Delhi India; 5 Department of Chemical and Bio-Technology, National Institute of Technology, Arunachal Pradesh, India; 6 Department of N.M.R., All India Institute of Medical Sciences, New Delhi, Delhi India; 7 Department of Chemistry, Indian Institute of Technology, Kharagpur, West Bengal India; 8 Institute of Post Graduate Medical Education & Research, Obstetrics & Gynecology, Kolkata, West Bengal India; 9 Institute of Reproductive Medicine, Sector-III, Kolkata, West Bengal India; 10 Department of Electronics & Communication Engineering, DSPM-IIIT, Naya Raipur, CG India

Correction to: *Scientific Reports* 10.1038/s41598-018-23954-7, published online 24 April 2018

In the original version of this Article, Mainak Dutta was incorrectly affiliated with ‘Department of Biotechnology, Birla Institute of Technology Pilani (Dubai Campus), Dubai, United Arab Emirates’. The correct affiliation is listed below.

Department of Biotechnology, Birla Institute of Technology and Science, Pilani (Dubai Campus), Dubai, United Arab Emirates.

This error has now been corrected in the PDF and HTML versions of the Article, as well as in the Supplementary Information file.

