# Supplementary material for: Metabolomics reveals perturbations in endometrium and serum of minimal and mild endometriosis
Source: Sci Rep. 2018 Apr 24;8:6466. doi: 10.1038/s41598-018-23954-7 (PMC5915433; doi:10.1038/s41598-018-23954-7)
Supplement: Supplementary file 1 — Supplementary Information [file 41598_2018_23954_MOESM1_ESM.pdf]

Metabolomics reveals perturbations in endometrium and serum of minimal and mild endometriosis

Mainak Dutta[1,2], Brajesh Singh[1], Mamata Joshi[3], Debanjan Das[1,10], Elavarasan Subramani[1], Meenu Maan[4], Saikat Kumar Jana[5], Uma Sharma[6], Soumen Das[1], Swagata Dasgupta[7], Chaitali Datta Ray[8], Baidyanath Chakravarty[9], Koel Chaudhury[1]

[1]*School of Medical Science and Technology, Indian Institute of Technology, Kharagpur, West Bengal, India*

[2]*Department of Biotechnology, Birla Institute of Technology and Science, Pilani (Dubai Campus), Dubai, United Arab Emirates*

[3]*National Facility for High-field NMR, Tata Institute of Fundamental Research, Mumbai, Maharashtra, India*

[4]*School of Biotechnology, Jawaharlal Nehru University, New Delhi, Delhi, India*

[5]*Department of Chemical and Bio-Technology, National Institute of Technology, Arunachal Pradesh, India*

[6]*Department of N.M.R., All India Institute of Medical Sciences, New Delhi, Delhi, India*

[7]*Department of Chemistry, Indian Institute of Technology, Kharagpur, West Bengal, India*

[8]*Institute of Post Graduate Medical Education & Research, Obstetrics & Gynecology, Kolkata, West Bengal, India*

[9]*Institute of Reproductive Medicine, Sector-III, Kolkata, West Bengal, India*

[10]*Department of Electronics & Communication Engineering, DSPM-IIIT, Naya Raipur, CG, India*

Supplementary Information

Identification of metabolites: Each chemically distinct moieties having distinct hydrogen nucleus in each metabolite exhibit an NMR signal at a characteristic resonance frequency, which is measured as a chemical shift relative to a standard compound. The exact chemical shift of the NMR signal of a hydrogen nucleus in a metabolite is precisely characteristic of that nucleus, in that metabolite, in the particular matrix conditions. In addition to chemical shift, another feature which is used for metabolite identification is spin multiplicity. Peaks arising from equivalent spin system protons split into more than one peak (multiplets: doublets (d), double doublets (dd), triplet (t), quartet (q)) due to chemically in-equivalent neighboring protons. This effect of spin-spin splitting is transmitted through bonds and is applicable only when the two nuclei are very close (maximum distance is three bonds) in the bonding network. The distance between the peaks in a given multiplet in a measure of the magnitude of splitting effect. It is referred to as coupling-constant and is independent of the applied field strength and depend only upon the molecular structure. Spin multiplicity thus provides vital information in determining neighboring protons. The method allowed manual identification of several metabolites from diverse chemical classes. Individual metabolites were also further verified from various sources, including earlier published articles, literatures and cross checked from the Human Metabolome Database (HMDB). Also, peak assignment was validated with COSY (Correlation spectroscopy) and TOCSY (Total correlation spectroscopy) spectra. The list of identified metabolites along with chemical shift and the type of multiplicity detected is provided in Supplementary Table 1.

Supplementary Table 1: List of metabolites detected in endometrial tissue extract using NMR

**Metabolites**	**Moieties**	**^1H ppm (multiplicity)$^\#$**	**Identified in**
3-hydroxybutyric acid	γCH_3, ½αCH_2, ½αCH_2	1.20 (d), 2.31 (dd), 2.41 (dd)	^1H, TOCSY, COSY
Acetic acid	CH_3	1.92 (s)	^1H
Adenine	2-CH, 6-CH	8.19 (s), 8.21 (s)	^1H, TOCSY, COSY
Alanine	βCH_3, αCH	1.5 (d), 3.79 (m)	^1H, TOCSY, COSY
Asparagine	CH_2, CH	2.94 (m), 4.0 (m)	^1H, TOCSY, COSY
Aspartic acid	½ βCH_2, ½ βCH_2, αCH	2.68 (dd), 2.82 (m), 3.89 (m)	^1H, TOCSY, COSY
Choline	N-$(CH_3)_3$, βCH_2, αCH_2	3.2 (s), 3.51 (t), 4.05 (t)	^1H, TOCSY, COSY
Citric acid	½ CH_2, ½ CH_2	2.55 (d), 2.69 (d)	^1H, TOCSY, COSY
Creatine	CH_3, CH_2	3.05 (s), 3.93 (s)	^1H, TOCSY, COSY
Formic acid	CH	8.47(s)	^1H
Glutamic acid	αCH, γCH_2	3.75 (m), 2.36 (m)	^1H, TOCSY,

Metabolites	Moieties	^{1}H ppm (multiplicity)[#]	Identified in
			COSY
Glutamine	βCH$_2$, γCH$_2$, αCH,	2.15 (m), 2.46 (m), 3.77 (m)	^{1}H, TOCSY, COSY
Glycine	αCH	3.58 (s)	^{1}H
Glycogen	1-CH	5.4 (m broad)	^{1}H
Inosine	8-CH, 14-CH	8.2 (s), 8.3 (s)	^{1}H, TOCSY, COSY
myo-Inositol	H1/H3 CH, H4/H6 CH	3.48 (dd), 3.6 (t)	^{1}H, TOCSY, COSY
Isoleucine	δCH$_3$, βCH$_3$, γCH$_2$, βCH, αCH	0.94(t), 1.02(d), 1.46 (m), 1.98 (m), 3.68 (d)	^{1}H, TOCSY, COSY
Lactic acid	βCH$_3$, αCH	1.35(d), 4.13(q)	^{1}H, TOCSY, COSY
Leucine	δCH$_3$, γCH, αCH,	0.96 (d), 1.7 (m), 3.73 (t),	^{1}H, TOCSY, COSY
Lysine	γCH$_2$, δCH$_2$, βCH$_2$, εCH$_2$, αCH	1.48 (m), 1.73 (m), 1.91 (m), 3.03 (t), 3.76(t)	^{1}H, TOCSY, COSY
Methionine	δCH$_3$, βCH$_2$, γCH$_2$	2.14 (s), 2.16 (m), 2.65 (t)	^{1}H, TOCSY, COSY
Nicotinurate	4-CH, 2-CH	8.25 (d), 8.92 (s)	^{1}H, TOCSY, COSY
Ornithine	δCH$_2$, αCH	3.08 (m), 3.74(m)	^{1}H, TOCSY, COSY
Phenylalanine	Ring-CH	7.35 (m), 7.40 (t), 7.45 (m)	^{1}H, TOCSY, COSY
Proline	CH$_2$, CH	2.08 (m), 3.4 (m)	^{1}H, TOCSY, COSY
Succinic acid	CH$_3$	2.39 (s)	^{1}H
Taurine	CH$_2$-SO$_3$, CH$_2$-NH	3.26 (t), 3.42 (t)	^{1}H, TOCSY, COSY
Threonine	αCH, βCH	3.59(d), 4.24 (m)	^{1}H, TOCSY, COSY
Tyrosine	CH, CH	6.92 (d), 7.21 (d)	^{1}H, TOCSY, COSY
Uracil	CH, CH	5.8 (d), 7.5 (d)	^{1}H, TOCSY, COSY
Uridine	12-CH, 11-CH	5.88 (d), 7.88 (d)	^{1}H, TOCSY, COSY
Valine	γCH$_3$, γ'CH$_3$, αCH, βCH	0.99 (d), 1.05 (d), 3.62 (d), 2.28 (m)	^{1}H, TOCSY, COSY
α-Glucose	1-CH	5.24 (d)	^{1}H, TOCSY, COSY
β-Glucose	1-CH	4.66 (d)	^{1}H, TOCSY, COSY
Glucose & mixed amino acids	(αCH)-resonances	3.3-3.9	^{1}H, TOCSY, COSY

[#]Key: s: singlet; d: doublet, dd: doublet of doublets; t: triplet; q: quartet; m: multiplet

Supplementary Table 2: Model parameters of the supervised models

**Model parameters (Tissue)**		**Stage I**	**(Stage II)**	**(Stage III)**	**Stage IV**
**PLS-DA**	R2X (Cum)	0.208	0.176	0.168	0.269
	R2Y (Cum)	0.943	0.992	0.903	0.981
	Q2 (Cum)	0.669	0.83	0.707	0.933
**OPLS-DA**	R2X (Cum)	0.208	0.176	0.168	0.227
	R2Y (Cum)	0.942	0.992	0.903	0.964
	Q2 (Cum)	0.692	0.835	0.729	0.93

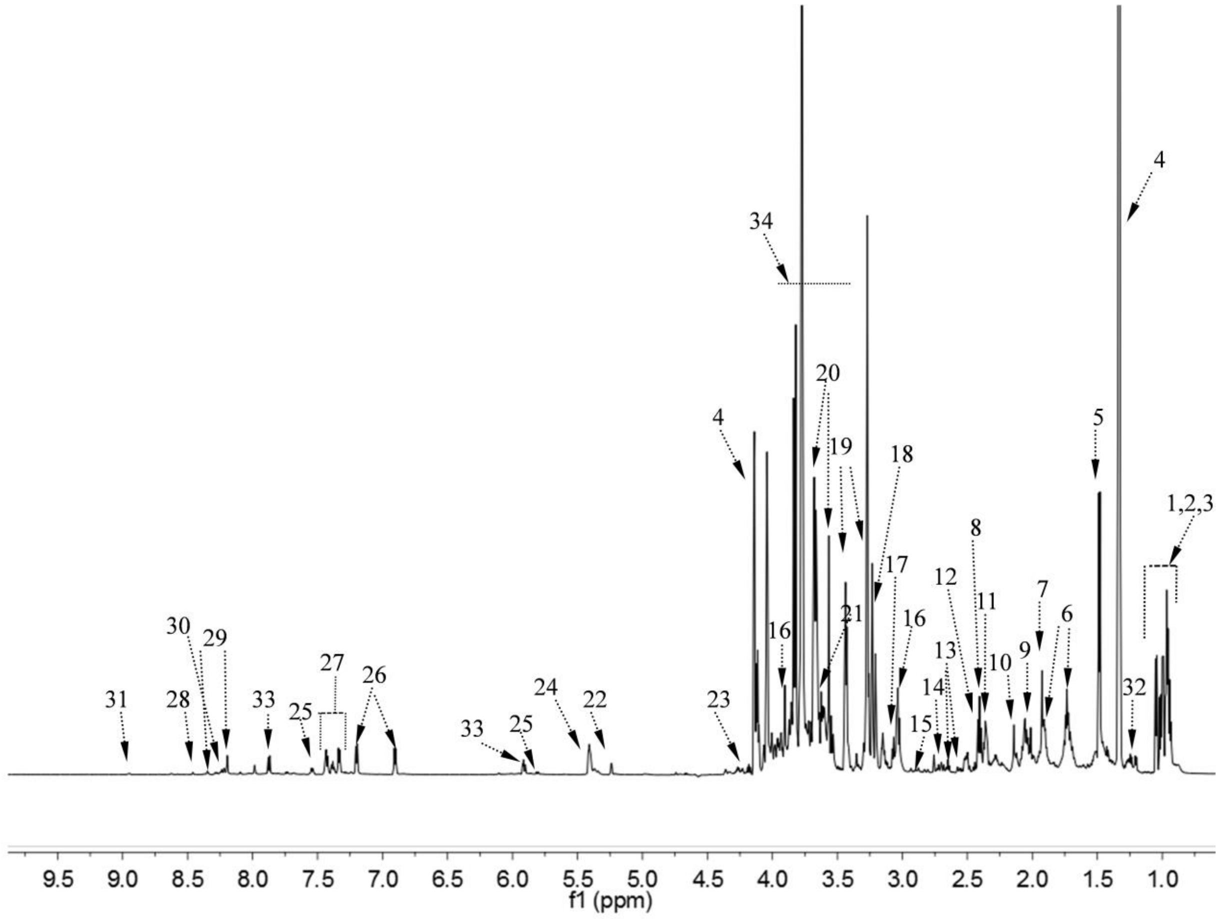

Supplementary figure 1: A representative 1H NMR spectra of eutopic endometrial tissue from an endometriosis patient. Leucine; 2: Isoleucine; 3:Valine; 4: Lactate; 5: Alanine; 6:Lysine; 7: Acetate; 8: Succinate; 9: Proline; 10: Methionine; 11: Glutamate; 12: Glutamine; 13: Citrate; 14: Aspartate; 15: Asparagine; 16: Creatine; 17: Ornithine; 18: Choline; 19: Taurine; 20: *myo*-inositol; 21: Glycine; 22: α Glucose; 23: Threonine; 24: Glycogen; 25: Uracil; 26: Tyrosine; 27: Phenylalanine; 28: Formate; 29: Inosine; 30: Adenine; 31: Nicotinurate; 32: 3-hydroxybutyrate; 33: Uridine; 34: Glucose and mixed amino acids (α CH) resonances

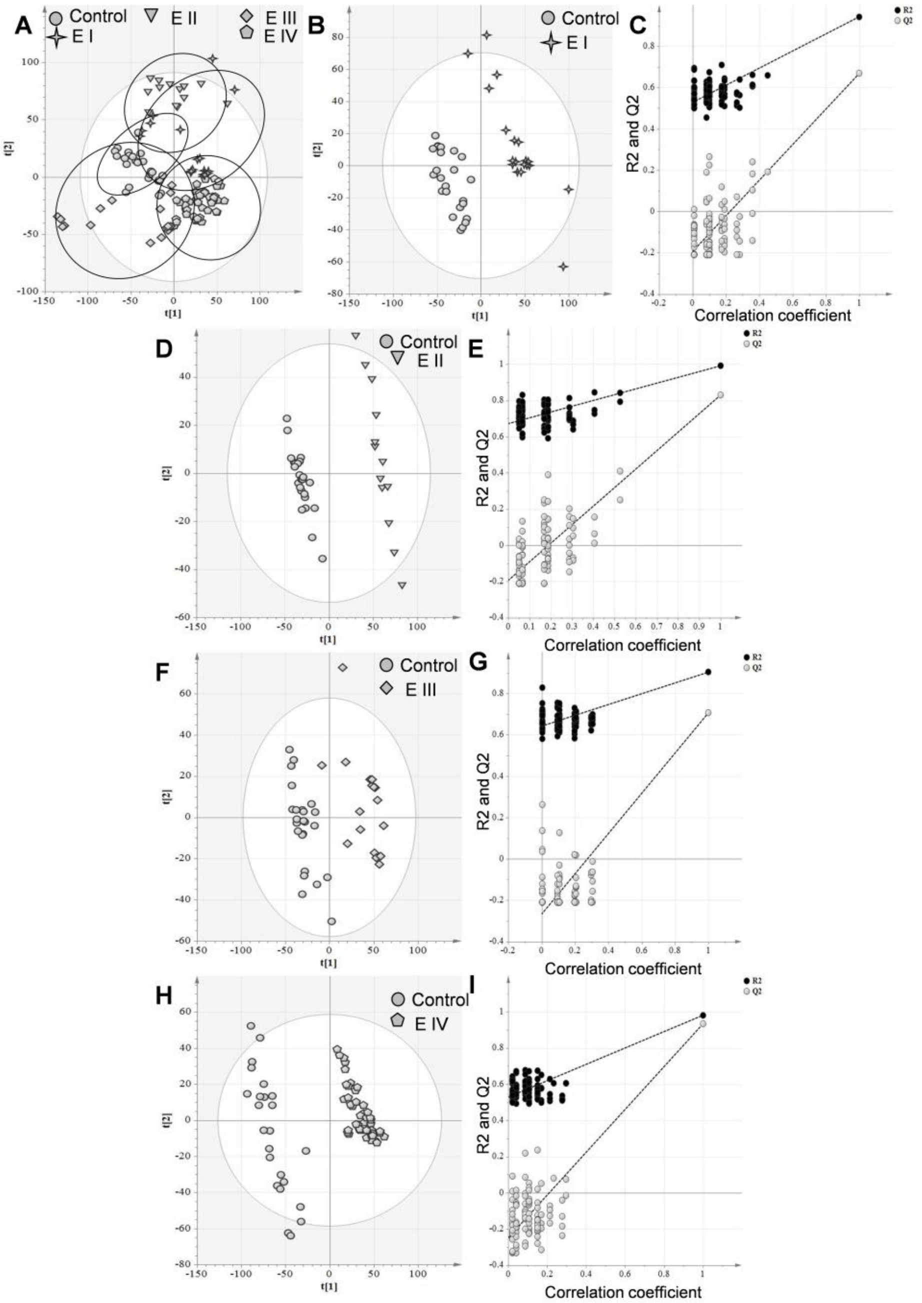

Supplementary figure 2: PCA and PLS-DA analysis of NMR spectra generated with tissue samples. PCA analysis of (A) all stages of endometriosis and controls. PLS-DA analysis of (B) control vs minimal endometriosis (E I), (D) control vs mild endometriosis (E II), (F) control vs moderate endometriosis (EIII), (H) control vs severe endometriosis (EIV). Permutation test statistics for the PLS-DA models of (C) control vs E I with Y-axis intercepts: R2 (0.0, 0.51), Q2 = (0.0, − 0.21), (E) control vs E II with Y-axis intercepts: R2 (0.0, 0.68), Q2 = (0.0, − 0.19), (G) control vs EIII with Y-axis intercepts: R2 (0.0, 0.66), Q2 = (0.0, − 0.26), (I) control vs EIV with Y-axis intercepts: R2 (0.0, 0.52), Q2 = (0.0, − 0.25).

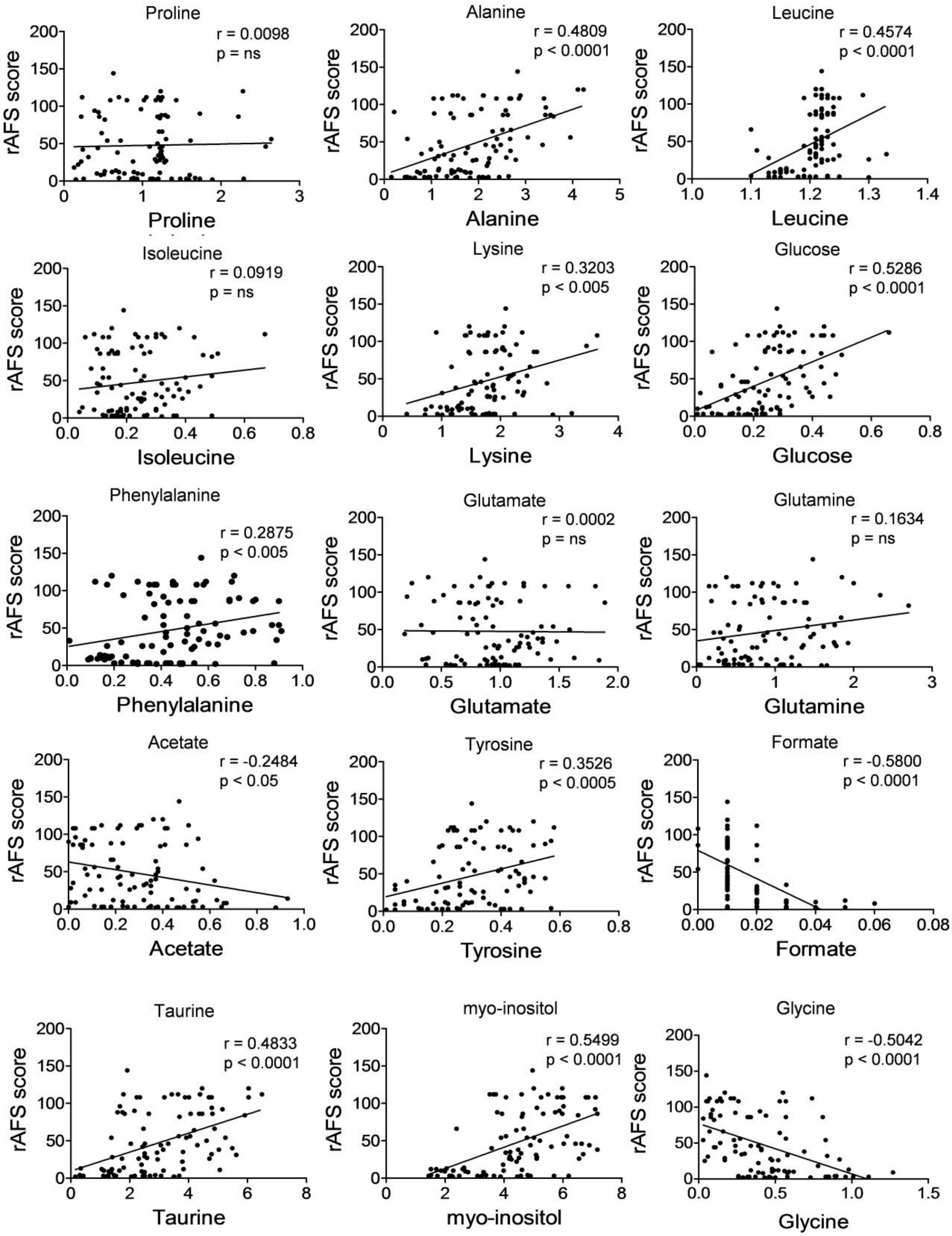

Supplementary figure 3: Correlation analysis between relevant tissue metabolites and rAFS score of women with endometriosis. Nonparametric Spearman correlation test was used
